# Globalism versus Nationalism in Medical Physics

**DOI:** 10.1002/acm2.12097

**Published:** 2017-05-15

**Authors:** Scott Dube, Jeroen B. van de Kamer, Yi Rong

**Affiliations:** ^1^ Department of Radiation Oncology Morton Plant Hospital Clearwater FL USA; ^2^ Department of Radiation Oncology Netherlands Cancer Institute Antoni van Leeuwenhoek Amsterdam The Netherlands; ^3^ Department of Radiation Oncology University of California Davis Comprehensive Cancer Center Sacramento CA USA

## 1 INTRODUCTION

Nowadays we medical physicists are bombarded with guidelines and task reports, and we may see similar ones from different organizations in the United States of America or worldwide. Many extraordinary and diligent medical physicists have devoted their time and knowledge in creating these documents. Yet some of us might be wondering: should we optimize the use of our resources by establishing a global medical physics society? One global medical physics organization may eliminate redundancies and improve cost‐effectiveness. However, it may also bring in inefficiency, lack of diversity, or poor environmental adaptation.

Our debate topic in this issue is: A global medical physics organization in science, education, professional, and administrative structures will result in greater advancement of the medical physics profession.

Arguing for the proposition is Scott Dube. Mr. Scott Dube is a solo medical physicist at Morton Plant Hospital in Clearwater, FL. He is also on the faculty of Radiological Technologies University and enjoys teaching aspiring medical physics and medical dosimetry students.

Arguing against the proposition is Jeroen van de Kamer. Mr. Jeroen van de Kamer is a medical physicist working at the Netherlands Cancer Institute at the department of radiation oncology. Together with his colleagues, he is involved with linac and patient‐specific QA, the clinical use of PET/CT, and the continuous development of the treatment for head and neck cancer. Jeroen is chair of the Netherlands Commission on Radiation Dosimetry, a Dutch–Belgium consortium of scientist aiming to promote the appropriate use of dosimetry of ionizing radiation. He was course director of the 2016 pre‐meeting ESTRO course “Multidimensional dosimetry systems.” Jeroen is a member of the advisory board of the Dutch Metrology Institute VSL.

## 2 OPENING STATEMENT

### 2.A Mr. Scott Dube

One of the leading controversies today is globalism versus nationalism. Globalism is based on all nations working together for the betterment of the planet. Nationalism is based on a single nation acting independently to pursue its best interest. The two ideologies have clashed on issues such as trade, immigration, human rights, climate change, and so on. In some ways, the practice of radiotherapy has been globalized by vendors such as Accuray, BrainLab, Elekta, Philips, RaySearch, Varian, and others who have created products which are standardized regardless where they are used.

There are also organizations striving to globalize the practice of medical physics. The oldest would be the International Atomic Energy Agency (IAEA) created in 1957. The IAEA maintains the Dosimetry Laboratory in Austria where Member States send their secondary reference dosimeters to ensure that measurement results are consistent with the International System of Units (SI). They also offer cost‐free safety guides, safety standards, and training material at the IAEA Radiation Protection of Patients (RPOP) website to provide professionals and patients worldwide with current information on the use of radiation in medicine. And there are other organizations. The International Organization of Medical Physics (IOMP) claims to represent 16 000 medical physicists worldwide. It has members from a number of national organizations and multinational federations, i.e., European Federation of Organizations for Medical Physics (EFOMP). It holds the Medical Physics World Congress every 3 years, and, in between, holds the International Conference on Medical Physics (ICMP). In addition, there are several organizations currently providing support to developing countries. Medical Physicists Working Beyond (MPWB) promotes access to physics‐based healthcare worldwide. RadiatingHope is providing training and equipment to developing countries. Their mission is to improve cancer care, specifically radiation oncology care, around the globe. The MD Anderson Dosimetry Laboratory supports their cause by calibrating donated dosimetry equipment at their ADCL and providing training via WebEx or video download. The International Medical Physics Certification Board (IMPCB) is promoting certification mechanisms in developing countries. They are helping with development of certification boards in countries or regions of countries so medical physicists can have recognition there.

At the same time, there is a strong sense of nationalism in the world of medical physics. The IOMP website lists 50 national medical physics organizations and six regional organizations. Many of these organizations have their own set of Task Groups and Committees which develop protocols, procedures, and practice guidelines for their own use. Such efforts are not trivial and require considerable time and resources typically provided by volunteers. For example, there are presently six independent protocols to define the calibration procedure for teletherapy beams: DIN 6800‐2 in Germany, IPEM in the UK, NCS 18 in the Netherlands, ÖNORM S 5234‐03 in Austria, TG‐51 in the USA and Canada, and IAEA TRS 398 in all other countries. Each is designed to ensure the same goal that radiation therapy units produce 1 Gy per 100 MU under calibration conditions. This is a clear example of nationalism.

Imagine how it would look under globalism. There would be a single Task Group which would create the calibration protocol to be followed by all nations. Globalization of a calibration protocol could be easily achieved if all nations agreed to follow IAEA TRS‐398. Its title speaks for itself, “Absorbed Dose Determination in External Beam Radiotherapy: An International Code of Practice for Dosimetry Based on Standards of Absorbed Dose to Water.” It was published in 2000 and its authors include physicists from Belgium, Germany, Italy, Japan, New Zealand, and the USA.

I believe that the success of globalism would provide benefits such as the following. First, it would eliminate the unproductive duplication of efforts by so many. Thousands of hours would be spared worldwide and could be redirected to more productive activities. Rather than several organizations reinventing the wheel, they could use a common wheel and invent other products which are truly necessary. Second, by having a single protocol for all nations to follow, it would eliminate the possibility of inconsistencies in the results among the many independent protocols. One can argue that these mismatches are not likely to occur because the independent national standards laboratories do crosscheck their protocols with each other and make adjustments as necessary. But this is additional time and effort which could be better spent elsewhere. Third, global acceptance of a single protocol would promote global collaboration. It would likely lead to other shared reports on topics such as for IMRT/VMAT quality assurance, implanted cardiac device precautions, small field dosimetry, and others. Once again this would increase the productivity of medical physicists everywhere by eliminating duplication of efforts.

Time is a most precious resource in the lives of individuals as well as the endeavors of nations. I believe it is in the best interest of all to follow globalism when developing protocols and practice standards. Imagine what could be accomplished when so many have more time available to address the limitless opportunities in medical physics.

### 2.B Mr. Jeroen van de Kamer

History has made it clear that diversity drives progress. In the fair and constant battle between competing technologies, new insights and creative ideas are being born, giving rise to a new cycle of competing technologies and so on. This virtuous circle is the fuel on which scientific advancement thrives.

It is widely known that most ground‐breaking ideas are not accompanied by naked persons shouting out “Eureka” but by thoughtful scientists thinking “Hmm… That's funny!” when examining results. In short, new ideas come from unexpected outcomes (“errors”) and us giving critical thoughts to these. Everybody should be allowed to learn from their errors, preferably in discussion with others. On top of that, one tends to understand the advantages and shortcomings of new wheels better if one is actively involved in the invention. This implies that everybody should try to invent one's own wheel, while still working together; cooperation does not mean that everybody should do the same thing.

This line of reasoning is easily translated to the development of dosimetry protocols: medical physics experts (MPEs) setting up a new protocol as a group gain a deeper understanding of the ins and outs of the protocol, along with its weaknesses and hidden assumptions. This in contrast to MPEs just following a recipe set by an international body. Introducing a newly formulated protocol and validating its implementation through site visits and audits should be practical, ergo done locally. A local implementation also assures optimal embedding in home‐grown customs, existing procedures, and available equipment. If one were to design a protocol that everybody should be able to conform to, it may be too simplistic for some and too laborious for others. Different levels of expertise demand dissimilar implementations. What works for some would not work for others, for example, due to accessibility of equipment or budgetary issues. Besides, being actively involved in setting up a new dosimetry protocol assures a thorough knowledge of dosimetry and calibration; a great way to learn!

But there are more advantages to setting up competing dosimetry protocols. The scientific struggle for life gives rise to a Darwinian competition, resulting in the survival of the fittest dosimetry protocols. Those who decided not to participate in the dosimetry rat race can choose the protocol that best suits their needs. This is how it should be in a free‐ and open‐minded thought‐provoking world. As explained above, this scientific competition between different protocols renders new ideas resulting in progress in the field of radiation dosimetry and standardization. Indeed, standardization benefits from diversity.

That being said, it remains important to compare the different dosimetry protocols and make sure that we speak the same language when reporting on dosimetry, a noble task for among others the various metrology and standard laboratories. While we are on the subject of standard laboratories, these laboratories all have their own, often homemade primary standards measuring “their” Gy. This diversity and redundancy is a must since different approaches have different strong and weak points. For example, primary standards based on graphite calorimeters complement the weaker points of water calorimeters and vice versa, resulting in a strong and robust system of dosimetry standards. These standards are compared in international comparisons, which prevent erroneous reading in a single standard laboratory to go undetected. If only one laboratory would define the Gy, such errors may go undetected and propagate all over the world and more.

Finally, all physicists know Newton's second law, which states that greater mass has a greater amount of inertia. Although it is commendable to have international or even worldwide consensus on dosimetry and quality assurance protocols, having a large body slows down the process and hinders technological progress. Locally formulated protocols are better suited to react on the ever‐changing demands on dosimetry imposed by new treatment techniques delivered by flattening filter free devices, such as Tomotherapy, Gammaknife, CyberKnife, MR‐guided devices, or particle therapy.

In short, setting up local protocols is quicker, is better suited to specific needs, and provides the best education for those involved. Dissemination of this knowledge is most efficient when done locally. Let us beware of the one ring to rule them all.

## 3 REBUTTAL

### 3.A Mr. Scott Dube

My admirable adversary said, “On top of that, one tends to understand the advantages and shortcomings of new wheels better if one is actively involved in the invention.” One definition of the word “protocol” is “a procedure for carrying out a scientific experiment or a course of medical treatment.” In other words, a protocol is prescriptive. It does not matter how well the nuances of the procedure are understood. Calibrating a linear accelerator involves making measurements and applying factors which are not meant to be deviated. It's a simple as that.

He also said, “The scientific struggle for life gives rise to a Darwinian competition, resulting in the survival of the fittest dosimetry protocols.” That sounds compelling but we know it is not true. The fittest as well as the weakest dosimetry protocol will survive regardless of the competition. That is because nationalism has allowed individual countries to choose their protocol without restriction.

And he also said, “If only one lab would define the Gy, such errors may go undetected and propagate all over the world and more.” That may be true. But which paradigm creates the greatest opportunity for error: Six committees developing six different protocols or one committee developing the protocol and the other five committees evaluating the same protocol? And which would be the most efficient use everyone's time? We must keep in mind that much of the work required to develop new protocols is done by volunteers. They must balance their time with the demands of their employer such as clinical duties, quality assurance, program development, teaching, research, and other activities which the employer expects of the physicist. The incentive for performing such work is not just job security but also service to the patients.

It is often said that organizations such as American Association of Medical Physicists (AAPM) are in need of volunteers to participate in Task Groups and Committees. As members, we have a duty to do what we can to help. But the duty should be two ways. By that I mean organizations have a duty to use the volunteers in an efficient and productive manner. To ask for volunteers to develop and maintain calibration protocols which have been duplicated by other organizations is a misuse of the volunteer's time. We are fortunate that so many physicists in so many countries have volunteered so much of their time to develop so many calibration protocols. But does that make it right that they do so? I believe they deserve to be relieved of redundant work. Developing and managing a single global dosimetry protocol would do that for many.

### 3.B Mr. Jeroen van de Kamer

Let me first compliment my esteemed opponent with his compelling plea. It is refreshing to hear sound arguments in favor of globalism these days, in general a good idea. However, in this case, the plea for globalism in the field of reference dosimetry is at fault, as I will show below.

The main argument for allowing only the One Protocol is, according to my opponent, to save time; well, except for the experts that take upon themselves this Herculean task. Even Task Groups of AAPM may take over 5 years, imagine what happens if a worldwide task group is set to work. But also suggesting that mundane physicist such as myself will save time this way is an honest but false promise. True, the time not spent in the glorious effort to devise the One Protocol can be spent in other ways, but we must not forget our duties: one of them is to implement the One Protocol as envisaged by the global team. This means reading it, trying to understand the choices made, probably based on hidden assumptions, and deciding how to put the protocol into effect in the clinic. It is a no‐brainer that many questions do arise during this process, potentially leading to the undesired inconsistencies in the implementation that my opponent rightfully warns against. Now we can try to bother the original authors with our humble questions, but they are probably too busy doing other important stuff they had to postpone while writing the One Protocol. So, no luck there. And since no responsible medical physics expert is willing to put a procedure into effect that he/she does not understand fully, there is not much left but consulting fellow physicists struggling with the same problem. This always has been and will be the pleasant burden for the early adaptors among us.

But wait, what happens if you get together front‐running physicists to discuss the One Protocol? They are bound to discover inconsistencies and ambiguities, and they will find smarter solutions, better suited to their needs. This already smells like a local protocol, better in line with local habits, requirements, and circumstances so why not jot it down for the benefit of others? The One Protocol will inescapably result in multiple local protocols, just by trying to implement it. Although my opponent refers to this as “unproductive,” it is just our job.

What is more problematic is that not all regions develop at the same speed. So what is needed and possible for one is impractical or not adequate for another. For example, dosimetry for flattening filter free systems, as those described in my opening statement, cannot be performed as demanded by the One Protocol since there is no flattened region over a 10 × 10 cm^2^ surface. Should we, therefore, stop using such clinical devices?

And while it is commendable that RadiatingHope and MPWB offer to help implementing the latest One Protocol, what happens after they have left? If the state‐of‐the‐art One Protocol is not in the genes of the local physicists, the problems will be larger than adhering to older, local dosimetry protocols that are tailored to the local level of expertise. I am convinced that the bottom‐up approach is better: let the local physicists decide when it is time to revise dosimetry protocols or setup IMRT/VMAT QA protocols; let them choose which way to implement it, either by copying a published protocol that is closest to their needs or by devising their own.

Helping each other to obtain the best possible results is highly commendable but please do not tell others what to do. There is no need to put the One Protocol into Mordors Mount Doom but the exclusive nature of it should.

